# Quantum Chaos in the Extended Dicke Model

**DOI:** 10.3390/e24101415

**Published:** 2022-10-04

**Authors:** Qian Wang

**Affiliations:** 1 CAMTP-Center for Applied Mathematics and Theoretical Physics, University of Maribor, SI-2000 Maribor, Slovenia; qwang@zjnu.edu.cn; 2 Department of Physics, Zhejiang Normal University, Jinhua 321004, China

**Keywords:** quantum chaos, extended Dicke model, spectral statistics, eigenstate structure

## Abstract

We systematically study the chaotic signatures in a quantum many-body system consisting of an ensemble of interacting two-level atoms coupled to a single-mode bosonic field, the so-called extended Dicke model. The presence of the atom–atom interaction also leads us to explore how the atomic interaction affects the chaotic characters of the model. By analyzing the energy spectral statistics and the structure of eigenstates, we reveal the quantum signatures of chaos in the model and discuss the effect of the atomic interaction. We also investigate the dependence of the boundary of chaos extracted from both eigenvalue-based and eigenstate-based indicators on the atomic interaction. We show that the impact of the atomic interaction on the spectral statistics is stronger than on the structure of eigenstates. Qualitatively, the integrablity-to-chaos transition found in the Dicke model is amplified when the interatomic interaction in the extended Dicke model is switched on.

## 1. Introduction

In recent years, the study of quantum chaos in many-body systems has attracted much attention, both theoretically and experimentally in different fields of physics, such as statistical physics [1,2,3,4,5], condensed matter physics [6,7,8,9,10,11,12,13], and high-energy physics [14,15,16,17,18,19], as well as quantum information science [20,21,22,23,24,25,26]. To some extent, this great interest in quantum many-body chaos is due to the close connections of chaos to several fundamental questions that arise in current theoretical and experimental studies. Although a full understanding of quantum many-body chaos is still lacking, much progress has been achieved. It is known that chaos in interacting quantum many-body systems can lead to thermalization [1,2,3], the fast scrambling of quantum information [14,27,28,29], and an exponential growth of quantum complexities [18,30,31,32,33,34,35], and diffusive transport [36].

The notion of chaos in the classical regime is usually defined by the so-called butterfly effect, namely the exponential separation of infinitesimally nearby trajectories for initial perturbations [37,38]. However, as the concept of trajectory is ill-defined in quantum mechanics, the definition of quantum chaos remains an open question. Therefore, probing the signatures of chaos in quantum many-body systems becomes a central task in the studies of quantum many-body chaos. To date, many complementary detectors of quantum chaos and the limits of their usefulness have been widely investigated in the literature [28,31,32,33,39,40,41,42,43,44,45,46,47,48,49,50,51,52,53,54]. Important model systems in this context are billiards [40,55]. Another task, which has recently also drawn great interest, is to unveil different factors that affect the chaotic properties of quantum many-body systems. While the impacts of the strength of disorder and the choice of initial states on the development of quantum chaos in various many-body systems have been extensively explored [4,56,57,58,59,60,61], more works are required in order to obtain deeper insights into the universal aspects of quantum many-body chaos.

In the present work, we analyze the emergence of chaos in the extended Dicke model. There are several different versions of the extended Dicke model [62,63,64,65]. Here, we focus on the one that has been discussed in ref. [63]. Different from the original Dicke model [66], which consists of an ensemble of noninteracting two-level atoms interacting with a single bosonic mode, the atoms in our considered extended Dicke model are permitted to interact. This allows us to analyze the effects of the atomic interaction on the degree of chaos of the model. Previously, the role of the atom–field coupling in the Dicke model for the emergence of quantum chaos has been investigated [67,68,69,70], while here we explore how this transition is affected by additional atomic interaction. By performing a detailed analysis of the energy spectral statistics and the structure of eigenstates, we systematically study both the chaotic signatures of the extended Dicke model and examine the effect of the atomic interaction on the chaotic features in the model. We show how the atomic interaction affects the spectral statistics and the structure of eigenstates, respectively.

The article is structured as follows. The model is introduced in Section 2. The influences of the atomic interaction on the energy spectral statistics is discussed in Section 3. A detailed investigation of the consequence of the atomic interaction on the structure of eigenstates is presented in Section 4. Finally, we conclude in Section 5 with a brief summary of our results and outlook.

## 2. Extended Dicke Model

As an extension of the original Dicke model [66,67,68], the extended Dicke model studied here consists of *N* mutual interacting two-level atoms with energy gap $\omega_{0}$ coupled to a single cavity mode with frequency ω. By employing the collective spin operators $J_{x,y,z}=\sum_{i=1}^{N}{\hat{\sigma }}_{x,y,z}^{\left(i\right)}$ (${\hat{\sigma }}_{x,y,z}$ are the Pauli matrices), the Hamiltonian of the extended Dicke model can be written as (hereafter, we set ℏ=1) [63,71]

(1) $$H=\omega a^{†}a+\omega_{0}J_{z}+\frac{2\lambda }{\sqrt{N}}J_{x}\left(a+a^{†}\right)+\frac{\kappa }{N}J_{z}^{2},$$

where $a(a^{†})$ denotes the bosonic annihilation (creation) operator, λ is the coupling strength between atom and field, and κ represents the strength of the atomic interaction.

The conservation of total spin operator $J^{2}=J_{x}^{2}+J_{y}^{2}+J_{z}^{2}$ for the Hamiltonian (1) leads to the Hamiltonian matrix being block diagonal in $J^{2}$ representation. In this work, we focus on the maximum spin sector j=N/2, which involves the experimental realizations and includes the ground state. Moreover, the commutation between Hamiltonian (1) and the parity operator $\Pi =e^{i\pi (j+J_{z}+a^{†}a)}$ enables us to further separate the Hamiltonian matrix into even- and odd-parity subspaces. Here, we restrict our study to the even-parity subspace.

To numerically diagonalize the Hamiltonian (1), we work in the usual Fock–Dicke basis {|n,m〉}={|n〉⊗|j,m〉}. Here, |n〉 are the Fock states of bosonic mode with n=0,1,2,…,∞, and |j,m〉 represent the so-called Dicke states with m=−j,−j+1,…,j. Then, the elements of the Hamiltonian matrix are given by

(2) $$\begin{array}{cc} \langle n^{\prime },m^{\prime }|H|n,m\rangle =\; & \left(n\omega +m\omega_{0}\right)\delta_{n^{\prime },n}\delta_{m^{\prime },m}+\frac{\kappa }{N}m^{2}\delta_{m^{\prime },m}+\frac{\lambda }{\sqrt{N}}\left[\sqrt{n}\delta_{n^{\prime },n-1}+\sqrt{n+1}\delta_{n^{\prime },n+1}\right]\\ \; & \times \left[\sqrt{j(j+1)-m(m+1)}\delta_{m^{\prime },m+1}+\sqrt{j(j+1)-m(m-1)}\delta_{m^{\prime },m-1}\right]. \end{array}$$

We remark that the value of *n* is unbounded from above, the actual dimension of the Hilbert space is infinite, regardless of the value of *j*. In practice, we need to cut off the bosonic number states at a larger but finite value $N_{c}$. Moreover, the dependence of the chaoticity in the Dicke model on the energy [63,72,73] further implies that it is also necessary to cut off the energy in order to obtain the finite number of considered states. In our numerical simulations, we set $N_{c}=320$ and restrict our analysis to the eigenstates with energies E/N∈[0.4,4]; the convergence of our results is carefully examined. For our selected energy interval, we checked that our main results still hold for other choices of $N_{c}$, as long as N≥16 and the convergence of the Fock–Dicke basis is fulfilled.

The extended Dicke model exhibits both ground-state and excited-state quantum phase transitions and displays a transition from integrable to chaotic behavior with increasing system energy, such as in the original Dicke model. The features of these transitions have been extensively investigated in the semiclassical regime [63]. It is worth mentioning that several possible experimental realizations of the extended Dicke model have been pointed out in [63,71,74].

## 3. Energy Spectrum Statistics

In this section, we explore the transition from integrability to chaos in the extended Dicke model by analyzing its energy-level spacing distribution. In our study, we focus on the energy levels with energies changing from E/N=0.4 to E/N=4. We compare our results to the level distributions of fully integrable and chaotic cases, respectively. We are aiming to characterize the quantum signatures of chaos in the model and unveil the impact of the atomic interaction on its chaotic properties.

### 3.1. Level Spacing Statistics

As the most frequently used probe of quantum chaos, the distribution P(s) of the spacings *s* of the consecutive unfolded energy levels quantifies the degree of correlations between levels. For integrable systems, where the energy levels are allowed to cross, the distribution P(s) is given by the Poissonian distribution [75],

(3) $$P_{P}\left(s\right)=\exp\left(-s\right).$$

On the other hand, the energy levels in chaotic systems exhibit level repulsion, and the distribution P(s) follows the Wigner–Dyson distribution [39,40,41]. For the systems with symmetric and real Hamiltonian matrices, as in the extended Dicke model, the Wigner–Dyson distribution has the following expression:

(4) $$P_{WD}\left(s\right)=\frac{\pi s}{2}\exp\left(-\frac{\pi }{4}s^{2}\right).$$

**Figure 1:**
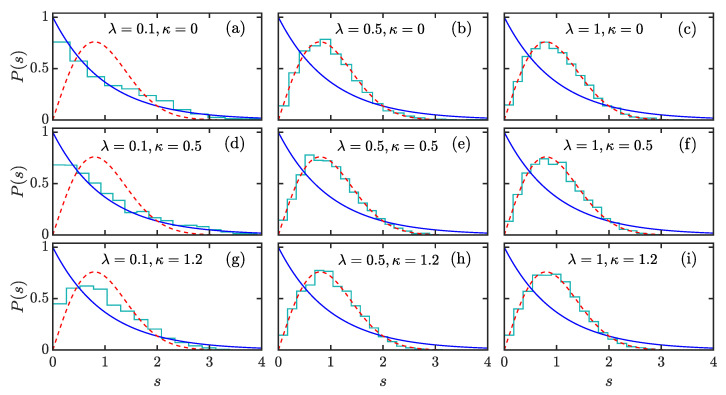
Level spacing distribution of the extended Dicke model for several combinations of λ and κ, see the legends in panels (**a**–**i**). The considered energy levels are the ones that have energies E/N∈[0.4,4]. The total atom number is N=2j=32, and the cut off in bosinic Hilbert space is $N_{c}=320$. The Poissonian (blue solid lines) and Wigner–Dyson distributions (red dashed lines) are, respectively, plotted in each panel for comparison. Other parameters are: $\omega =\omega_{0}=1$. All quantities are dimensionless.

In Figure 1, we show P(s) of the extended Dicke model with $\omega =\omega_{0}=1$ and j=N/2=16 for different values of λ and κ. Here, the level spacings *s* are obtained from the unfolded eigenlevels ${\tilde{E}}_{\mu }=E_{\mu }/\Delta E$, with 1/ΔE being the local density of states. One can clearly see that P(s) undergoes a transition to Wigner–Dyson distribution as λ increases, regardless of the value of κ. The case κ=0 is the original Dicke model as in our previous work [68]. However, we find that, with increasing κ, the Poissonian distribution at λ=0.1, and κ=0 in Figure 1a turns into an intermediate case, as observed in Figure 1g. This suggests that the degree of chaos in the extended Dicke model can be tuned by the atomic interaction.

To quantitatively characterize the effect of the atomic interaction on the degree of chaos, we consider the proximity of P(s) to Wigner–Dyson or to Poissonian distributions. There are several ways to measure the distance between two distributions, here, the difference between P(s) and $P_{WD}\left(s\right)\left[P_{P}\left(s\right)\right]$ is quantified by the chaos indicators η and β. The indicator η is defined as [7,67,76]

(5) $$\eta =\left|\frac{\int_{0}^{s_{0}}\left[P\left(s\right)-P_{WD}\left(s\right)\right]ds}{\int_{0}^{s_{0}}\left[P_{P}\left(s\right)-P_{WD}\left(s\right)\right]ds}\right|,$$

where $s_{0}=0.4729\ldots$ is the first intersection point of $P_{WD}\left(s\right)$ and $P_{P}\left(s\right)$. For $P\left(s\right)=P_{WD}$, we have η=0, while $P\left(s\right)=P_{P}\left(s\right)$ leads to η=1. The indicator β is the level repulsion exponent and can be obtained by fitting P(s) to the Brody distribution [77]

(6) $$P_{B}\left(s\right)=b_{\beta }\left(\beta +1\right)s^{\beta }\exp\left[-b_{\beta }s^{\beta +1}\right],$$

where the factor $b_{\beta }$ is given by

(7) $$b_{\beta }={\left[\Gamma \left(\frac{\beta +2}{\beta +1}\right)\right]}^{\beta +1},$$

with Γ(x) being the gamma function. The value of β varies in the interval β∈[0,1]. When β=0, it means the level spacing distribution P(s) is Poisson. On the other hand, for chaotic systems, we would expect $P\left(s\right)=P_{WD}\left(s\right)$, and therefore β=1.

**Figure 2:**
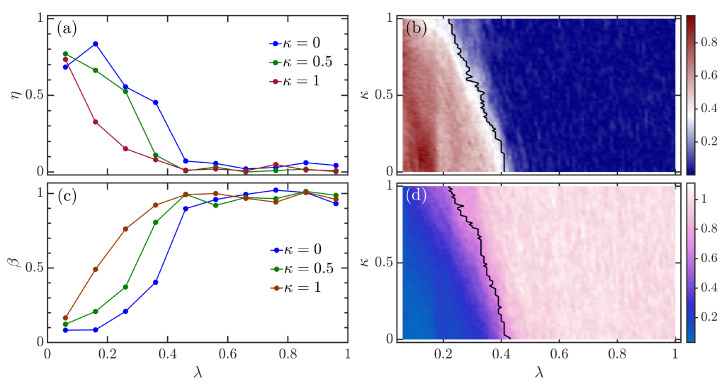
(**a**) Chaos indicator η as a function of λ for several values of κ. (**b**) η as a function of κ and λ. (**c**) Chaos indicator β as a function of λ for different values of κ. (**d**) β as a function of κ and λ. These results are obtained from the energy levels with energies E/N∈[0.4,4]. In panels (**b**,**d**), the boundaries of the chaotic region are marked by the black curves, which are defined as η≤0.3 and β≥0.7, respectively. Other parameters are: $\omega =\omega_{0}=1$ and N=2j=32. All quantities are dimensionless.

In Figure 2a, we plot η as a function of λ for various values κ. It can be seen that irrespective of the strength of the atomic interaction, the extended Dicke model undergoes a transition from integrability to chaos as the coupling strength λ increases. However, as the strength of the atomic interaction increases, the onset of chaos happens for smaller values of coupling strength λ. This is more evident from Figure 2b, where we show how η evolves as a function κ and λ. Clearly, the width of the region with larger values of η decreases with increasing κ, suggesting that the location of the crossover to quantum chaos moves towards the lower values of λ with increasing κ. The statement above is further confirmed by the boundary of chaotic region, which is plotted as the black curve in Figure 2b. Here, we determine the boundary of chaos by the condition $\eta \leq \eta_{d}=0.3$ [76]. We set the threshold $\eta_{d}=0.3$ as it implies that the model has already departed from the integrablity and is tending to the chaotic regime.

Figure 2c shows β as a function of λ with increasing κ. As observed in Figure 2a, while the chaotic behavior at higher values λ is independent of κ, the coupling strength λ that needs to be for the transition to chaos decreases with increasing κ. Figure 2d illustrates β as a function of κ and λ. One can see that with increasing κ, the region with β≈1 extends to smaller values of λ. By identifying the chaotic region as β≥0.7, we show that the boundary of chaos strongly depends on the strength of the atomic interaction, see the black curve in Figure 2d. Notice that the boundary extracted from η behaves in a similar way to the one extracted from β.

### 3.2. Level Spacing Ratio

The study of level spacing distribution requires the so-called unfolding procedure [78]. It proceeds by rescaling the original eigenlevels to ensure that the local density of states of the resulting spectrum is 1. It is usually a nontrivial task, in particular for quantum many-body systems. To circumvent this disadvantage, one can resort to another chaotic probe based on the ratio of adjacent level spacings [79], which is free from the unfolding procedure.

For a given set of level spacings $\left\{s_{\mu }=E_{\mu +1}-E_{\mu }\right\}$, the ratio of adjacent level spacings is defined as [79,80]

(8) $$r_{\mu }=\min\left(\delta_{\mu },\frac{1}{\delta_{\mu }}\right),$$

where $\delta_{\mu }=s_{\mu +1}/s_{\mu }$ is the ratio between two adjacent level spacings. Obviously, $r_{\mu }$ is defined in the interval $r_{\mu }\in \left[0,1\right]$. The distribution of $r_{\mu }$ for both integrable and chaotic systems has been analytically investigated [80,81,82]. It is known that for the chaotic systems with Hamitonian from the Gaussian orthogonal ensemble (GOE), the level spacing ratio distribution is given by

(9) $$P_{GOE}\left(r\right)=\frac{1}{Z_{1}}\frac{2(r+r^{2})}{{\left(1+r+r^{2}\right)}^{5/2}},$$

where $Z_{1}=8/27$ is the normalization constant. On the other hand, as the eigenlevels in the integrable systems are uncorrelated (independent Poisson levels), one can simply find that the ratio distribution is

(10) $$P_{Poi}\left(r\right)=\frac{2}{{\left(r+1\right)}^{2}}.$$

Due to $r_{\mu }\in \left[0,1\right]$, the ratio distribution $P_{GOE/Poi}\left(r\right)$ vanishes outside the range [0,1].

**Figure 3:**
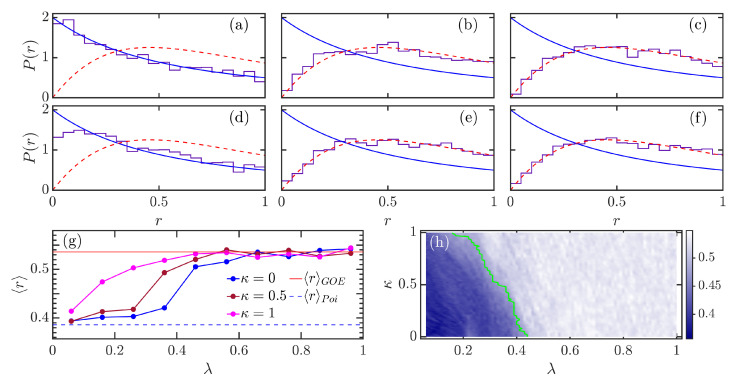
Level spacing ratio distributions P(r) for various combinations of κ and λ: (**a**) κ=0,λ=0, (**b**) κ=0,λ=0.5, (**c**) κ=0,λ=1, (**d**) κ=0.7,λ=0, (**e**) κ=0.7,λ=0.5, and (**f**) κ=0.7,λ=1. In each panel, the Poisson distribution $P_{Poi}\left(r\right)$ is plotted as a blue solid curve, while the red dashed line denotes $P_{GOE}\left(r\right)$. (**g**) Averaged level spacing ratio 〈r〉 as a function of λ for several values of κ. (**h**) 〈r〉 as a function of κ and λ. The green line indicates the chaotic boundary, which is determined by 〈r〉≥0.48. The energy levels used in our numerical calculation have energies E/N∈[0.4,4]. Other parameters: $\omega =\omega_{0}=1$ and N=2j=40. All quantities are dimensionless.

Figure 3a–f show how the level spacing ratio distribution P(r) evolves for different combinations of atomic interaction strength κ and coupling strength λ. Similarly to what we observe for the level spacing distribution P(s) in Figure 1, the spacing ratio distribution P(r) tends to $P_{GOE}$ with increasing coupling strength λ, independent of κ value. However, as evident from Figure 3a,d, increasing κ leads to the enhancement in degree of chaos of the model at smaller values of λ. Therefore, as mentioned above, the atomic interaction can be used to tune the level of chaoticity in the model. By switching on the interatomic interaction κ>0, the regularity-to-chaos transition of the original Dicke model [68,70] is amplified.

A more stringent analysis of the effect of the atomic interaction is made with the average level spacing ratio, defined as

(11) $$\langle r\rangle =\int_{0}^{1}rP\left(r\right)dr.$$

It takes the value ${\langle r\rangle }_{Poi}=2\ln2-1\approx 0.386$ for integrable systems with $P\left(r\right)=P_{Poi}\left(r\right)$, while for chaotic systems with $P\left(r\right)=P_{GOE}\left(r\right)$, one has ${\langle r\rangle }_{GOE}=4-2\sqrt{3}\approx 0.536$. Hence, 〈r〉 acts as a detector to diagnose whether the studied system is in the integrable or chaotic regime and has been widely used to track the crossover from integrability to chaos.

Figure 3g demonstrates 〈r〉 as a function of λ for three different values κ. We see that, regardless of the value of κ, the transition from integrability to chaos is well captured by the behavior of 〈r〉, which varies from ${\langle r\rangle }_{Poi}$ to ${\langle r\rangle }_{GOE}$ with increasing λ. We further observe that the chaotic phase is robust with respect to the variation of κ, but the integrable phase exhibits a strong dependence on κ. For the integrable phase with smaller values of λ, we find that increasing κ gives rise to an increase in 〈r〉. As a consequence, the location of transition to chaos can be varied by the atomic intraction. This effect is more clearly observed in Figure 3h, where the evolution of 〈r〉 as a function of κ and λ is illustrated. Again, we define the boundary of chaotic region by the condition $\langle r\rangle \geq {\langle r\rangle }_{c}=0.48$, meaning that the region with value 〈r〉≥0.48 is considered as chaos. We checked that our main result still holds for other choices of ${\langle r\rangle }_{c}$, as long as ${\langle r\rangle }_{c}\in \left(0.45,0.5\right)$. The green line in Figure 3h denotes the obtained boundary of chaos. As expected, the behavior of the boundary line confirms the extension of the chaotic region to lower values of λ as κ increases.

## 4. Structure of Eigenstates

The onset of chaos also bears a remarkable change in the structure of eigenstates. In this section, we explore the impact of atomic interaction on the transition to chaos by investigating the variation in the structure of eigenstates.

It is known that the eigenstates of chaotic systems are uncorrelated and are well described by random matrix theory (RMT) [3,83,84,85]. For the model studied in this work, one can expect that in the chaotic phase the eigenstates of the model will have the same structure as those of random GOE matrices. The GOE eigenstates are fully delocalized random vectors with real components consisting of independent Gaussian random numbers. Hence, the deviation of the eigenvector structure from Gaussian behavior is an alternative benchmark to certify quantum chaos [42,86,87,88,89].

The analysis of the structure of eigenstates requires expansion of the eigenstates in a chosen basis. The choice of basis is usually decided by the physical problem and the system under consideration. Here, we use the Fock–Dicke bases, {|n,m〉}, which are the eigenstates of $a^{†}a$ and $J_{z}$, as mentioned in Section 2. The decomposition of the *k*th eigenstate, |k〉, of the Hamiltonian (1) in the selected basis is given by

(12) $$|k\rangle =\sum_{\nu =1}^{D}c_{k}^{\nu }|\nu \rangle ,$$

where |ν〉=|n,m〉, D is the dimension of the Hilbert space, and $c_{k}^{\nu }=\langle \nu |k\rangle$ are the *k*th eigenstate components in the basis {|ν〉}, satisfying the normalization condition $\sum_{\nu }{\left|c_{k}^{\nu }\right|}^{2}=1$. The characterizations of the eigenstate structure are provided by the statistical properties of eigenstate coefficients $\left\{c_{k}^{\nu }\right\}$.

To analyze the fingerprint of chaos in the properties of the eigenstates of *H* in Equation (1), as well as to examine whether the atomic interaction has an effect on the eigenstate structure, we explore the coefficients’ distribution in comparison with the corresponding GOE result. As mentioned above, the eigenstates for the chaotic systems with real and symmetric D-dimensional Hamiltonian matrices are consistent with the GOE eigenstates. The components of a GOE eigenstate, $\left\{c_{\nu }\right\}$, are random numbers that are uniformly distributed on a unit sphere with dimension D−1. In the limit D≫1, the dependence between components vanishes, and the distribution of components can be well described by a Gaussian distribution with zero mean and variance 1/D [77,90,91,92],

(13) $$P_{GOE}\left(c\right)=\sqrt{\frac{D}{2\pi }}e^{-Dc^{2}/2}.$$

In the chaotic systems, it has been known that the coefficients of the mid-spectrum eigenstates are distributed as a near-Gaussian distribution [77,78,93,94,95], while the coefficients’ distribution for the eigenstates of nonchaotic systems and the edge eigenstates of chaotic systems is significantly different from Gaussian distribution [95,96,97]. As increasing λ leads to the onset of chaos in the model; one would expect that the distribution of mid-spectrum eigenstates coefficients should be turned from non-Gaussian into near-Gaussian.

**Figure 4:**
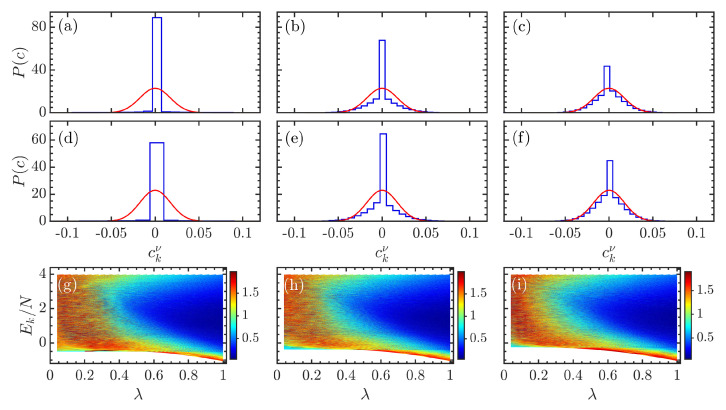
Panels (**a**–**f**): Histogramas of the coefficients $c_{k}^{\nu }$ of eigenstates with energy $E_{k}/N\in \left[1.75,2.25\right]$, in the Fock–Dicke basis. Each panel corresponds to different (κ,λ) combinations: (**a**) κ=0,λ=0, (**b**) κ=0,λ=0.5, (**c**) κ=0,λ=1, (**d**) κ=0.7,λ=0, (**e**) κ=0.7,λ=0.5, and (**f**) κ=0.7,λ=1. The red solid line in each panel denotes the Gaussian distribution in Equation (13). Panels (**g**–**i**): Kullback–Leibler divergence $D_{KL}$ [cf. Equation (14)] as a function of rescaled energy $E_{k}/N$ and λ for κ=0 (**g**), κ=0.5 (**h**), and κ=1 (**i**). Other parameters: $\omega =\omega_{0}=1$ and N=2j=40. All quantities are dimensionless.

Figure 4a–f show the evolution of the eigenstate coefficient distributions, denoted by P(c), for several (κ,λ) combinations in comparsion with the Gaussian distribution provided by Equation (13). We see that, regardless of the value of κ, the eigenstate coefficients’ distribution tends to Gaussian as λ increases. The larger the value of λ is, the higher the degree of chaos in the model, and, therefore, the closer the coefficients’ distribution to Gaussian, as expected. Another prominent feature, that is also independent of κ, observed in the behaviors of P(c) is the larger peak around $c_{k}^{\nu }∼0$. Even in the most chaotic regime, P(c) still exhibits a high peak near zero, as shown in Figure 4c,f. This excessive number of zero coefficients is mainly due to the mixed feature of the model. This means the regular and chaotic behavior coexist in the model for the considered parameters. Detailed analysis of the mixture of regular and chaotic behaviors in the extended Dicke model is beyond the scope of the present work; we leave this investigation for a future work.

Let us now turn to discuss how the atomic interaction κ affects the eigenstate coefficients’ distribution. As evident from Figure 4a–f, the presence of atomic interaction has almost no effect on the behavior of the eigenstate coefficients’ distribution, but just a reduction in the height of peak in the regular phase [compare Figure 4a to Figure 4d]. This implies that the impact of the atomic interaction on the structure of eigenstates is not as strong as on the eigenlevels.

To measure the difference between P(c) and Gaussian distribution (13), we consider Kullback–Leibler (KL) divergence [98], which is commonly used to measure how close an observed distribution is to a predicted distribution. In our study, P(c) and $P_{GOE}\left(c\right)$ are, respectively, identified as the observed and predicted distributions. Then, the KL divergence between them is given by

(14) $$D_{KL}=\int_{c_{min}}^{c_{max}}P\left(c\right)\ln\left[\frac{P(c)}{P_{GOE}\left(c\right)}\right]dc,$$

where $c_{min}$ and $c_{max}$ denote, respectively, the minimum and maximum values in $\left\{c_{k}^{\nu }\right\}$. The KL divergence is non-negative, so that $D_{KL}\geq 0$, and it vanishes only when $P\left(c\right)=P_{GOE}\left(c\right)$. Qualitatively, a larger $D_{KL}$ value indicates a larger difference between P(c) and $P_{GOE}\left(c\right)$.

In Figure 4g–i, we plot the KL divergence for the extended Dicke model as a function of $E_{k}/N$ and λ with κ={0,0.5,1}. We see that the behaviors of $D_{KL}$ versus $E_{k}/N$ and λ for different κ values are very similar. As expected, the eigenstates in the non-chaotic phase (λ≲0.45) and the eigenstates at the spectrum edge in the chaotic phase (λ≳0.5) have larger values of $D_{KL}$, suggesting that the corresponding coefficients’ distributions strongly deviate from Gaussian, as illustrated in Figure 4a,d for the integrable case. Here, it is worth pointing out that the infinite dimension of the Hilbert space for the extended Dicke model leads to its spectrum has only one edge, namely the ground state. On the other hand, the lower values of $D_{KL}$ for mid-spectrum eigenstates in the chaotic phase imply that their coefficients’ distributions are closer to Gaussian, as shown in Figure 4c,f. The similarity between behaviors of $D_{KL}$ observed in the bottom row of Figure 4 prompts a more detailed investigation of the impact of the atomic interaction on the eigenstate coefficient distribution.

**Figure 5:**
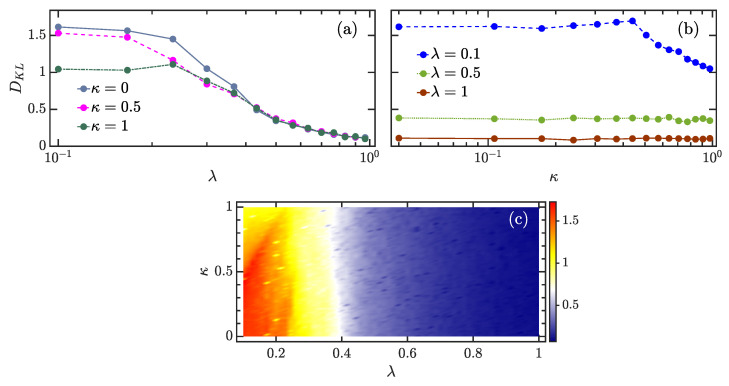
(**a**) KL divergence $D_{KL}$ (14) as a function of λ for several κ values. (**b**) $D_{KL}$ as a function of κ for several coupling strengths λ. (**c**) Color-scaled plot of $D_{KL}$ in the κ−λ plane. In all panels, the coefficients distribution P(c) is obtained from the eigenstates with energies $E_{k}/N\in \left[1.75,2.25\right]$. Other parameters: $\omega =\omega_{0}=1$ and N=2j=40. All quantities are dimensionless.

To determine whether the eigenstate coefficients’ distribution is robust with respect to the variation of the atomic interaction κ, we calculate $D_{KL}$ for the mid-spectrum eigenstates with energies $E_{k}/N\in \left[1.75,2.25\right]$. The final result for various cases is shown in Figure 5. The evolution of $D_{KL}$ as a function of λ for different values of κ is plotted in Figure 5a. One can see that the behavior of $D_{KL}$ for different κ is very similar. The KL divergence varies slowly for smaller λ until λ≈0.25, after which it rapidly decreases to small values as the coupling strength is increased beyond $\lambda_{c}\approx 0.5$. The change in $D_{KL}$ behavior is a manifestation of the transition to chaos resulting from the reduction in eigenstates correlation with increasing λ. We also observe that the dependence of $D_{KL}$ on κ in the integrable phase is very different from that of the chaotic phase. The explicit dependence of $D_{KL}$ on κ for several values of λ is shown in Figure 5b. As expected from Figure 5a, in the chaotic regime with λ≳0.5, $D_{KL}$ is independent of κ, whereas the KL divergence strongly depends on the atomic interaction for the cases of smaller λ. Overall, the KL divergence decreases with increasing κ in the integrable regime. This indicates that the proximity of the eigenstate coefficients’ distribution to the Gaussian distribution can be improved by the atomic interaction. Therefore, the atomic interaction can vary the degree of chaos in the extended Dicke model, in agreement with the results obtained from eigenvalue statistics. An overall evolution of $D_{KL}$ as a function of κ and λ is depicted in Figure 5c. We see that the chaotic region remains almost unchanged as κ increases, in contrast to the extension behavior revealed by the eigenvalue-based detectors of quantum chaos [see Figure 2b,d,h]. This suggests that the level repulsion is more sensitive to the effect of atomic interaction than the structure of eigenstates.

## 5. Conclusions

In this article, we performed a detailed analysis of quantum chaotic characters of the extended Dicke model through the statistical properties of eigenvalues and eigenstates. The presence of interaction between atoms in the model further allows us to explore the dependence of chaotic properties of the model on the atomic interaction. It was shown that the integrability-to-chaos transition of the original Dicke model as a function of the atom–field coupling is amplified by the interatomic interaction.

We demonstrated that as the model moves from regular phase to chaotic phase, both the level spacing and level spacing ratio distributions undergo a crossover from Poisson to Wigner distribution, regardless of the strength of atomic interaction. However, the presence of atomic interaction can lead to a notable deviation of level spacing and spacing ratio distributions from Poisson distribution. To quantify this deviation and to measure the degree of chaos in the model, we consider three different chaos indictors to probe the transition from integrablity to chaos. All of these indicators are complementary to each other and are able to capture the crossover from Poisson to Wigner for both level spacing and level spacing ratio distributions. We have also shown that the behaviors of these indicators as a function of control parameters are very similar. In particular, we found that the degree of chaos of the model can be controlled by tuning the strength of atomic interaction. This result highlights the role of interaction in the development of chaos in quantum many-body systems and opens up the possibility to tune the degree of chaos in the extended Dicke model.

Additional quantum chaotic signatures showing how the atomic interaction affects the degree of chaos in the extended Dicke model are unveiled in the structure of the eigenstates. To analyze the eigenstate structure, we expand each eigenstate in the Fock–Dicke basis and focus on the expansion coefficients’ distribution for mid-spectrum eigenstates. For fully chaotic systems with Hamitonian from GOE, such distribution is well described by Gaussian distribution. We showed that the transition to chaos can be detected by the deviation of coefficients’ distribution from Gaussian distribution. However, we note that even within the chaotic phase, the coefficients’ distribution is still different from Gaussian, indicating the existence of correlations between them. By using the KL divergence to measure the distance between the coefficients’ distribution and Gaussian distribution, we illustrated that the onset of chaos corresponds to the rapid decrease in the behavior of KL divergence as a function of coupling strength. Although the atomic interaction leads to the decrease in KL divergence in the regular phase, the transition to chaos revealed by KL divergence is almost independent of atomic interaction. This is different from the results obtained by the eigenvalue-based chaos indicators and implies that unlike the eigenlevels, the eigenstate structure is robust with respect to the change in atomic interaction.

A natural extension of the present work is to investigate the dynamical role played by the atomic interaction in the development of chaos. It would also be interesting to analyze the effect of atomic interaction on the level of chaoticity through long-range spectral correlations, which can be detected by the spectral form factor [41]. In addition, understanding the emergence of chaos and the impact of atomic interaction from the dynamics of the classical counterpart of the model would be another interesting topic. Very recently, the critical phenomena in the extended Dicke model have been thoroughly analyzed in this direction [65]. Finally, we would like to mention that a direct demonstration of level spacing distribution in an ultracold-atom system has been realized in a recent experiment [99]. Hence, we expect that the spectral statistics of our studied extended Dicke model can be verified by state-of-the-art experimental platforms. 

## Data Availability

Not applicable.
